# Identification of a *KEAP1* Germline Mutation in a Family with Multinodular Goitre

**DOI:** 10.1371/journal.pone.0065141

**Published:** 2013-05-28

**Authors:** Risa Teshiba, Tatsuro Tajiri, Kenzo Sumitomo, Kouji Masumoto, Tomoaki Taguchi, Ken Yamamoto

**Affiliations:** 1 Division of Genome Analysis, Research Center for Genetic Information, Medical Institute of Bioregulation, Kyushu University, Fukuoka, Japan; 2 Department of Pediatric Surgery, Graduate School of Medical Sciences, Kyoto Prefectural University of Medicine, Kyoto, Japan; 3 Department of Pediatric Surgery, Shimonoseki City Hospital, Shimonoseki, Japan; 4 Department of Pediatric Surgery, Faculty of Medicine, University of Tsukuba, Tsukuba, Japan; 5 Department of Pediatric Surgery, Reproductive and Developmental Medicine, Graduate School of Medical Sciences, Kyushu University, Fukuoka, Japan; Maastricht University Medical Center, The Netherlands

## Abstract

**Background:**

The familial clustering of multinodular goitres (MNGs) with a dominant mode of inheritance has been repeatedly reported. Linkage studies have revealed several genetic loci responsible for familial MNG; however, most of the causative variants remain unknown.

**Methods and Results:**

Through linkage analysis using single-nucleotide polymorphism markers, we identified a new MNG locus on 19p13.2-q12 in a five-generation Japanese MNG family. Subsequent mutation searches focusing on the candidate 25-Mb region of chromosome 19 identified a heterozygous mutation, c.879_880delinsA, p.Asp294Thr, fs*23, in exon 3 of the *KEAP1*, which plays a central role in the cytoprotection pathway against oxidative stress. Reverse transcriptase-PCR analysis showed low expression of wild type *KEAP1* accompanied by no transcription product of mutant allele in the normal and goitre region of thyroid tissues obtained from the proband. In agreement with previous studies showing that KEAP1 negatively regulates NFE2L2, the NFE2L2 target genes *GSTA4* and *GCLC* were up-regulated in the thyroid tissues of the patient.

**Conclusions:**

This study identified the first *KEAP1* mutation in MNG. The results provide insights into the pathogenesis of goitre which develops in the organ continuously exposed to oxidative stress during hormone synthesis.

## Introduction

A nontoxic multinodular goitre (MNG) (Online Mendelian Inheritance in Man [OMIM] 138800) is clinically characterised by the nodular enlargement of the thyroid gland without thyroid dysfunction or inflammation. In general population in an area with borderline iodine deficiency, MNG are found by ultrasonography in approximate 23% of the population [1]. Multiple factors, such as sex, iodine deficiency and smoking, influence the development of MNG [2]–[5]. In addition, familial and twin studies in endemic and nonendemic regions have indicated a genetic predisposition for MNGs [2], [5]–[7]. Because a history of MNG has been suggested to be a risk factor for the development of toxic goitres and thyroid cancer [8], [9], it is important to identify the genes involved in MNG for early diagnosis and for a better understanding of the pathogenesis of thyroid disease.

Linkage analysis using familial MNG has been one of the approaches used to identify causative genes for the disease. Several studies have reported genetically linked loci for familial MNG. Studies in German and Canadian pedigrees with nontoxic MNG identified a susceptibility locus on chromosome 14q, which is called MNG-1 (OMIM 138800) [10], [11]. In 2000, Capon et al. mapped MNG-2 (OMIN 300273) on chromosome Xp22 with linkage analysis of a three-generation Italian pedigree [12]. Another locus, MNG-3 (OMIM 606082), was identified on chromosome 3q26.1-q26.3 from two independent Japanese pedigrees presenting with MNG with euthyroidism and high thyroid-stimulating hormone levels [13]. Furthermore, four candidate loci were reported on chromosomes 2q, 3p, 7q and 8p with linkage analysis in Danish, German and Slovakian families [14]. Thus, linkage results indicate genetic heterogeneity in the aetiology of MNG, even in familial cases with a Mendelian mode of inheritance [15].

Based on observations that pleuropulmonary blastoma (PPB) families also have MNG, Rio Frio et al. screened mutations of the PPB-causative gene dicer 1, ribonuclease type III (*DICER1*), which is located in the MNG-1 locus in MNG families, and identified several mutations linked to MNG [16]. However, except for this recent finding, causative genes for euthyroid MNG in other loci have remained unclear. Considering the genetic heterogeneity of the disease, additional linkage analyses using additional families to validate reported loci or to detect novel loci followed by comprehensive sequencing analysis targeting linkage regions are required to explain the genetic aetiology of MNG.

In the present study, we identified a novel germline mutation in kelch-like ECH-associated protein 1 (*KEAP1*) in a Japanese pedigree with MNG through a combination of genome-wide linkage analysis using a high-density single-polymorphism nucleotide (SNP) array and next-generation sequencing. Subsequent gene expression analysis showed the functional activation of nuclear factor (erythroid-derived 2)-like 2 (NFE2L2), which is negatively regulated by KEAP1, in the thyroid tissue of the patient. Our study suggests that dysfunction of the KEAP1-NFE2L2 stress-response pathway may contribute to the nodular process in thyroid tissue, which is physiologically exposed to the oxidative nature of thyroid hormone synthesis.

## Methods

### Subjects

**Figure 1:**
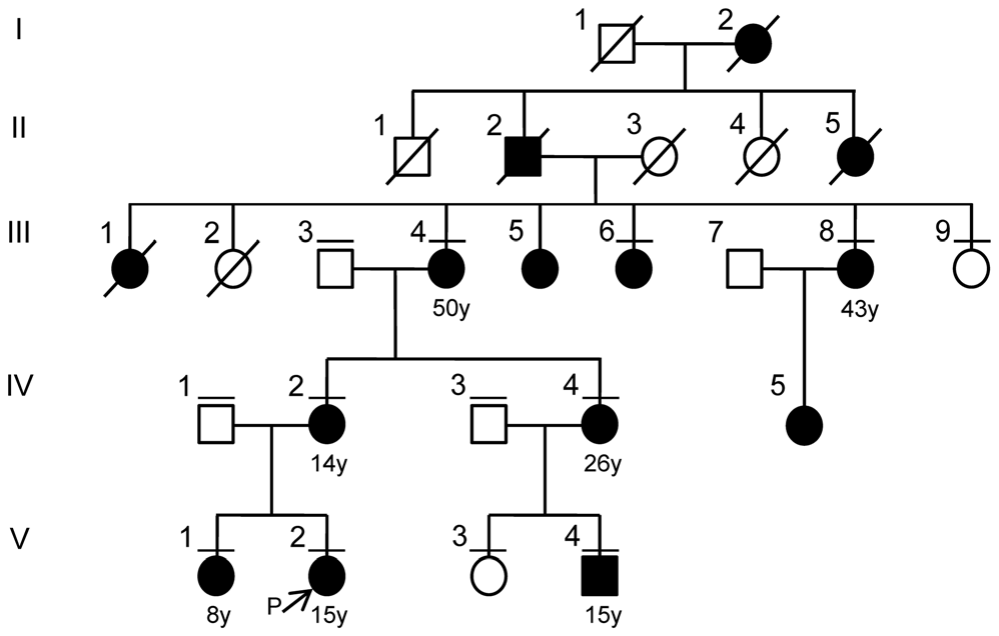
A pedigree of a five-generation multinodular goitre family. Individuals are numbered above each symbol. Individuals affected with MNG are indicated by filled symbols and age of onset is described under the patient symbol. The arrow shows a proband of this family study. The bars above each symbol indicate individuals included in the linkage analysis.

We identified a 5-generation Japanese family presenting with a dominant inheritance pattern of familial thyroid goitre (Figure 1 and Table S1). The proband (V∶2) was a 15-year-old girl from the western area of Japan who had been referred to our hospital. Ultrasonographic examination showed multiple cystic lesions in both of the thyroid lobes, and the level of thyroid hormone was within the normal range (TSH, 0.67 µU/ml; free T3, 4.16 pg/ml; free T4, 1.56 ng/dl). Her older sister (V∶1) had a thyroid lobectomy at 8 years old. Their pathological diagnoses were adenomatous goitre. They do not show any sign of malignancy in organs including the thyroid in medical follow-up after a thyroid lobectomy. We obtained 13 blood samples from the family, including 8 affected (III∶4, III∶6, III∶8, IV∶2, IV∶4, V∶1, V∶2 and V∶4) and 5 unaffected (III∶3, III∶9, IV∶1, IV∶3 and V∶3) individuals, and goitre and matched normal thyroid tissue from the proband (V∶2). The age of onset was 8–50 years old. None of the affected individual showed any evidence of malignancy. A total of 192 unrelated normal controls from a population-based Japanese cohort were subjected to sequencing to evaluate the frequency of the *KEAP1* mutation in the general population. All participants provided their written informed consent to participate in this study according to the Declaration of Helsinki. We obtained written informed consent from the parents on the behalf of the children participants. This study was approved by the Ethics Committee at Kyushu University, Fukuoka, Japan.

### DNA isolation and SNP genotyping

Genomic DNA was extracted from peripheral blood leukocytes using the QIAamp DNA Blood Midi Kit (Qiagen) and adjusted to a final concentration of 50 ng/µl for SNP typing. The Illumina Human CNV370K-Quad Array and the Illumina BeadStation 500G SNP genotyping system were employed for genome-wide SNP typing according to the manufacturer’s protocols. SNP genotypes were determined with the BeadStudio Genotyping Analysis Module 3.3.7 software. All of the samples showed call rates greater than 0.99 (the average was 0.998, Table S2), and 342,115 SNPs on chromosomes 1 to X had a call frequency of 1.0. Based on the results of the Mendelian error check of PLINK v1.06 (http://pngu.mgh.harvard.edu/purcell/plink/) [17], 293 SNPs were excluded, and the remaining 341,822 SNPs were available for linkage analysis. To estimate genome-wide pairwise IBD, we additionally excluded 94,826 SNPs that were homozygous in all of the samples; the remaining 246,996 SNPs were subjected to the IBD analysis. The quality control of the SNP typing is shown in Figure S1.

### Statistical analysis

The genome-wide IBD for all pairs of individuals was estimated using PLINK v1.06 to evaluate pedigree errors. Multipoint parametric LOD scores and the information score were calculated using GeneHunter v2.1r5 (with easyLINKAGEPlus v5.08) [18], [19]. A total of 3,514 and 454 SNPs whose pair-wise r-square was <0.01 were automatically selected by easyLINKAGE for linkage analysis to the whole genome (1.0 cM spacing) and to chromosome 19 (0.2 cM spacing), respectively (Table S3). The model used in the parametric analyses assumed a dominant model of inheritance, a disease allele frequency of 0.001 and complete penetrance. The marker genetic position was based on the Marshfield linkage map [20], and the sex-averaged position was applied.

### Exome capture and next-generation sequencing

The genomic DNA of the proband was subjected to sequencing. The SureSelect Human All Exon System (Agilent Technologies) was employed to capture whole-genome exons. The Illumina Genome Analyser GAIIx was used for sequencing, and 75 base reads were generated.

### Read mapping and variant analysis of the linkage region

Read mapping and the subsequent variant analysis of chromosome 19 were performed with CLC Genomic Workbench 4.6.1 (CLC bio). The following parameters were used to map reads to a reference genome (GRCh37): similarity  =  0.95, length fraction  =  1.0, insertion cost  =  3, deletion cost  =  3 and mismatch cost  =  2. Subsequently, to discover the conflict of a mapping, SNP and DIP (deletion/insertion polymorphisms) detections were performed using the default setting. To select potential mutations critical for the pathology of MNG, we eliminated the known variants that were listed in dbSNP132 or the variants that showed synonymous amino acid substitution. The reads that were incorrectly mapped were identified and removed using BLAT Search Genome in the UCSC database (http://genome.ucsc.edu/). The conflicts that resulted in a low quality value were considered to be artefacts. A summary of the sequencing and mapping is shown in Figure S2. Polyphen-2, Mutation Taster and SIFT classification tools were used to determine the amino acid changes that most likely destroyed protein function [21]–[23]. The direct sequencing of *KEAP1* was performed using the primers 5'- CTGAGCGACTGTCGGAAGTA-3' and 5'-GGCCAAGCAAGAGGAGTTCT-3' for PCR amplification and the primer 5'-GGCCAAGCAAGAGGAGTTCT-3' (the reverse primer of PCR) for sequencing according to a standard procedure.

### Reverse transcriptase-PCR

Total RNA was extracted from frozen thyroid tissues of the proband using an RNeasy Mini Kit (Qiagen) and was subjected to reverse transcription with the use of a First-strand cDNA Synthesis Kit (GE Healthcare). The resulting cDNA was then subjected to PCR analysis using 4 primer pairs: KEAP1-N-fwd, 5'-CCTGCAGTGACTTCCTGGTG-3' (forward); KEAP1-N-rvs, 5'-GTGGAAGACCTCGGACTCG-3' (reverse); KEAP1-C-fwd, 5'-ATGAGCGTGCCCCGTAAC-3' (forward); KEAP1-C-rvs, 5'-CACGGCATAAAGGAGACGAT-3' (reverse); KEAP1-mut-fwd, 5'-CATCTACATGCATTTTGGGGAG-3' (forward); KEAP1-mut-rvs, 5'-TAGTCCTTGCAGCGGGAGTT-3' (reverse); GAPDH-fwd, 5'-ACCACAGTCCATGCCATCAC-3' (forward); GAPDF-rvs, 5'-TCCACCACCCTGTTGCTGTA-3' (reverse). The KEAP1-mut-rev primer contained the "T" nucleotide at the 3' end to achieve specific amplification of mutated allele. The wild type full-length *KEAP1* cDNA and truncated *KEAP1* cDNA containing c.879_880delinsA mutation were cloned into *Sfi*I and *Xho*I sites of pCMV-myc plasmid (Life Technologies) for the control templates of PCR.

### Real-time PCR

The cDNA generated as mentioned above was also subjected to real-time PCR analysis in triplicate for evaluation of the expression of *NFE2L2* and its target genes glutathione S-transferase alpha 4 (*GSTA4*), glutamate-cysteine ligase catalytic subunit (*GCLC*) and NAD(P)H dehydrogenase quinone 1 (*NQO1*) with the use of a TaqMan Gene Expression Assay (Life Technologies) and the On ECO Real-time PCR System (Illumina), with glyceraldehyde-3-phosphate dehydrogenase (*GAPDH*) cDNA serving as an endogenous control. The following TaqMan probes were used: *NFE2L2* (Hs00232352_m1), *GSTA4* (Hs00155308_m1), *GCLC* (Hs00155249_m1) and *NQO1* (Hs00168547_m1). The amount of mRNA was calculated by the comparative CT method [24], and all data were normalized by the amount of *GAPDH* mRNA.

## Results

Genome-wide SNP typing enables the estimation of pairwise identity-by-descent (IBD) to examine the relationships of family members. Because pedigree error leads to incorrect results for linkage analysis, we first calculated the pairwise proportions of IBD in all of the samples and compared them with the theoretical values obtained from the pedigree chart. The genetic relationship of all of the pairs was consistent with the clinically obtained family relationship data (Figure S3); thus, linkage analysis was performed based on the original pedigree.

**Figure 2:**
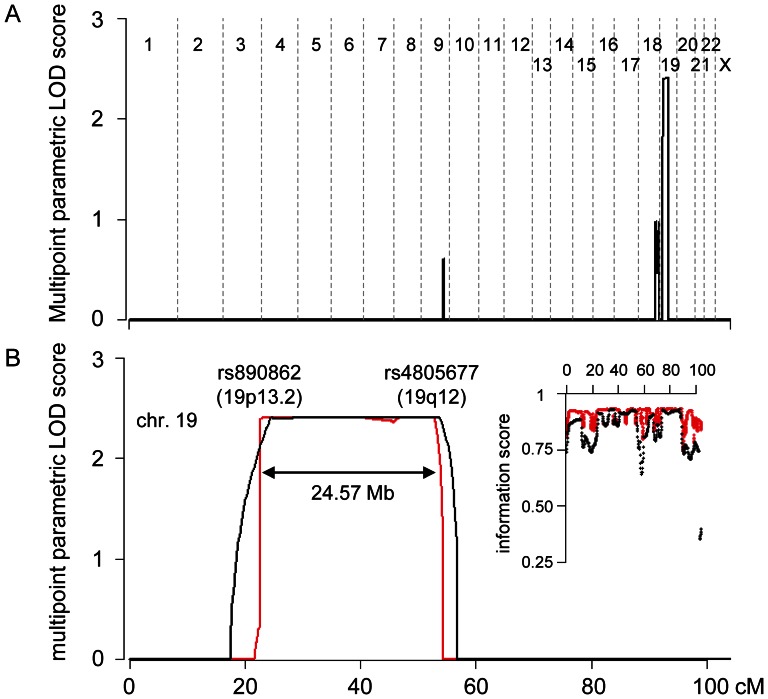
Summary of the genome-wide SNP linkage analysis. (A) The multipoint parametric LOD (pLOD) score with a marker interval of 1.0 cM is shown for all of the autosomes and the X chromosome. (B) The multipoint pLOD and the information score of chromosome 19 are shown. The black and red lines (or dot) denote results from 1.0 and 0.2 cM marker interval analysis, respectively.

The results of the multipoint linkage analyses using SNP markers are shown in Figure 2. A maximum parametric LOD (pLOD) score of 2.41 was obtained on chromosome 19, spanning approximately 25 Mb between rs890862 and rs4805677. The information scores approached 0.9 as the interval of the markers became narrower (Figure 2B), suggesting that analysis with a 0.2 cM marker interval was a more powerful test than a 1.0 cM interval. Linkage signals for the previously reported loci, MNG-1, -2 and -3 and chromosomes 2q, 3p, 7q and 8p, were almost zero (Figure 2A). The linkage analysis suggested that 19p13.2-q12 is a causative region in this family.

Three candidate genes were present within this linkage region: translocase of inner mitochondrial membrane 44 (*TIMM44*), NADH dehydrogenase (ubiquinone) 1 alpha subcomplex, 13 (*NDUFA13*) and solute carrier family 5 (sodium iodide symporter), member 5 (*SLC5A5*). Mutations in *TIMM44* and *NDUFA13* have been reported to be associated with tumorigenesis in the thyroid [25], [26], and the *SLC5A5* gene encodes sodium-iodide symporter protein, whose down-regulation is associated with thyroid carcinomas [27]. However, no mutation was identified in these genes in our samples (data not shown).

Considering the broad range of the linkage region, in which approximately 660 genes reside, we applied a next-generation sequencing technique for the identification of the causative mutation of MNG. The genomic DNA from a proband was subjected to exon capture, and a total of 263,794,206 reads with 75 bases were obtained with sequencing. A total of 5,314,723 reads were mapped to the reference sequence of chromosome 19 using *CLC* Genomics Workbench. The mean depth of coverage for the coding sequences was 92.1x, and 88.9% of the coding sequences on chromosome 19 were covered at greater than or equal to 1x depth. Within the exons and exon-intron boundaries of the linkage region, 7,222 single-nucleotide variations and 419 small deletions/insertions were detected with the SNP and DIP detection algorithms, respectively. After removing known variants, synonymous substitutions, systematic artefacts and coverage <10x, seven variants remained (Table 1 and Figure S2).

**Table 1:** Novel variants identified in the proband in the chromosome 19 linkage region.

Algorithm	Position of ref. on Chr19	Gene	Ref. base	Variant base	Count(ref./variant)	Frequency(ref./variant)	Amino acid change	Mutation taster	PolyPhen-2 v2.1.0		SIFT
									HumVar	evaluation	
DIP detection	10,602,698	*KEAP1*	C	-	27/28	49.1/50.9	frameshift	Disease causing	-	-	-
SNP detection	8,196,653	*FBN3*	C	T	0/11	0/100	Gly592Asp	Disease causing	1.000	Most likely damaging	Not scored
	9,068,277	*MUC16*	G	C	17/12	58.6/41.4	Ala6390Gly	Polymorphism	NA	Not predicted	Not predicted
	10,445,269	*ICAM3*	C	T	44/56	44.0/56.0	Ser376Asn	Polymorphism	0.006	Benign	Not scored
	11,943,552	*ZNF440*	G	A	101/97	51.0/49.0	Gly521Arg	Polymorphism	0.012	Benign	Tolerated
	16,613,993	*C19orf44*	C	T	76/77	49.7/50.3	Arg293Cys	NA	0.098	Benign	Tolerated
	21,239,988	*ZNF430*	G	A	159/151	51.3/48.7	Glu291Lys	Polymorphism	0.045	Benign	Tolerated

ref.: reference sequence.

The six missense variants and one deletion were evaluated to determine functionally significant changes using Mutant Taster (http://www.mutationtaster.org/), PolyPhen-2 (http://genetics.bwh.harvard.edu/pph2/) and SIFT (http://sift.bii.a-star.edu.sg/) programs [21]–[23]. Four variants located in *ICAM3*, *ZNF440*, *C19orf44* or *ZNF430* were assumed to lead to non-hazardous changes in protein function because at least two programs evaluated them as "polymorphism", "benign" or "tolerated" (Table 1). Indeed, the variants in *ICAM3*, *ZNF440* or *C19orf44* were recently identified by 1000 Genome Project (rs117942573, rs117998813 or rs199764040, respectively). Therefore, we excluded these four variants from further analysis. A variant in *MUC16* was evaluated as "polymorphism" by the Mutation Taster program, and expression of this gene in the thyroid was suggested to be considerably low based on several databases including BioGPS (http://biogps.org/), Cancer Genome Anatomy Project (http://cgap.nci.nih.gov/SAGE) and GeneHub-GEPIS (http://research-public.gene.com/Research/genentech/genehub-gepis/). These results suggested that the variant in *MUC16* could not be a significant candidate of causative mutation of the MNG. Regarding a variant in *FBN3*, all of the read contained the variant base, indicating that the genotype was homozygous for the variant. Therefore, it was unlikely that this variant causes the disease with dominant mode of inheritance. Finally, we selected a frame shift variant in *KEAP1* for further analysis because it was a novel variant, evaluated as "disease causing", and showed heterozygous genotype.

**Figure 3:**
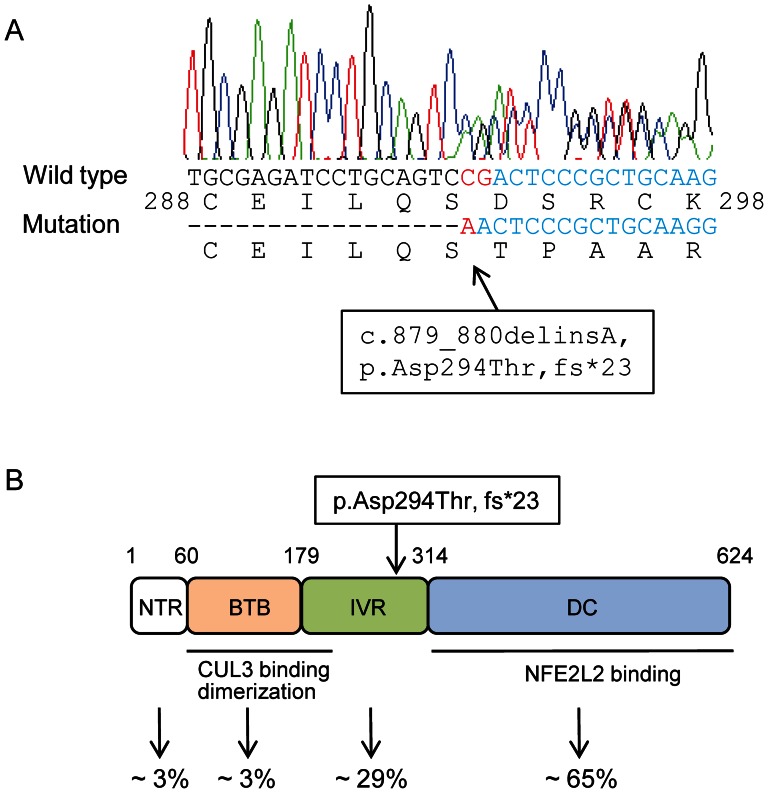
Identification of a heterozygous mutation in ***KEAP1*** **.** (A) The result of Sanger sequencing of proband DNA showed the c.879_880delinsA mutation (red), resulting in a 1-base deletion and a frameshift (p.Asp294Thr, fs*23) in *KEAP1*. (B) Domain structure and mutation location in the KEAP1 protein. The protein consists of an N-terminal region (NTR; amino acids 1 to 60), a BTB domain (amino acids 61 to 179), an intervening region (IVR; amino acids 180 to 314) and a DC domain (amino acids 315 to 624). The BTB and N-terminal portion of IVR is responsible for dimerisation and the interaction with CUL3. The DC domain is also critical for the interaction with NFE2L2. The p.Asp294Thr, fs*23 mutation is located in the IVR. The reported frequencies of the somatic mutations observed in each domain in cancer cells are shown at the bottom [32].

Direct sequencing of the *KEAP1* variant in the proband identified a frameshift mutation in exon 3 of the gene: c.879_880delinsA, p.Asp294Thr, fs*23 (Figure 3A). The subsequent sequencing of family members confirmed the co-segregation of the mutation in affected individuals (Figure S4). However, the mutation was not observed in 192 Japanese normal control samples.

**Figure 4:**
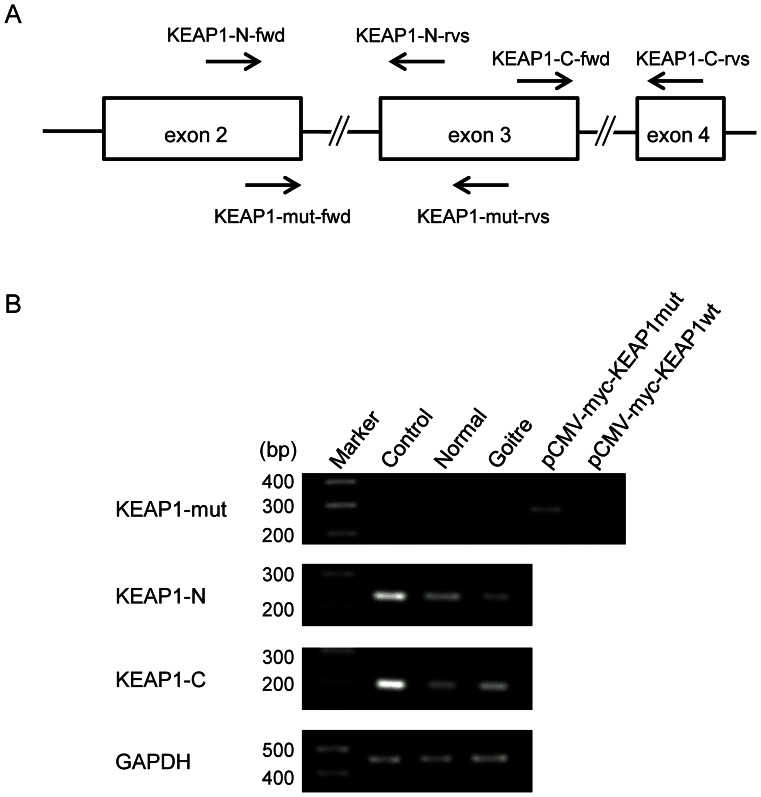
Reverse transcriptase-PCR for mutant and wild type ***KEAP1*** **.** (A) Primer positions for reverse transcriptase-PCR in KEAP1 exons. The asterisk indicates the position of c.879_880delinsA mutation in exon 3. The primer, KEAP1-mut-rvs, contains "T" nucleotide at the 3' end which is the complement of mutant "A" nucleotide to amplify mutant allele. The expected sizes of PCR products are as follows: 233 bp for KEAP1-N-fwd and KEAP1-N-rvs; 201 bp for KEAP1-C-fwd and KEAP1-C-rvs; 282 bp for KEAP1-mut-fwd and KEAP1-mut-rvs. (B) Results of the reverse transcriptase-PCR for the mutant and wild type *KEAP1*. cDNAs from control normal thyroid, the normal and goitre region of thyroid tissues obtained from the proband were amplified by 3 primer sets indicated in (A) to mutant *KEAP1* (upper panel), 5' and 3' normal portions adjacent mutation site (second and third panels, respectively) of *KEAP1*. *GAPDH* was amplified as a control for the amount of cDNA in each sample. The expected size of PCR product of *GAPDH* is 452 bp. In the experiment of mutant KEAP1 amplification, the plasmids containing mutant or wild type KEAP1 (pCMV-myc-KEAP1mut or pCMV-myc-KEAP1wt, respectively) were utilized as control templates.

KEAP1 is a cytoplasmic protein that acts as a negative regulator of NFE2L2 [28]–[30]. KEAP1 interacts with NFE2L2 via an DC domain comprising six Kelch repeats and a C-terminal region and also associates with CUL3 via a broad complex tramtrack bric-a-brac (BTB) homodimerisation domain to form an E3 ubiquitin ligase complex, resulting in the constitutive degradation of NFE2L2 (Figure 3B). Somatic mutations that impair the function of KEAP1 and lead to the constitutive stabilisation of NFE2L2 have been found in lung, gallbladder and liver cancer in humans (Figure 3B) [31], [32]. Because the p.Asp294Thr, fs*23 mutation identified in this study resides in a DC domain, we questioned whether this mutation could generate a truncated KEAP1 protein or not. We examined the expression of the mutant *KEAP1* in the thyroid tissues of the patient by reverse transcriptase-PCR using 3 primer pairs (Fig 4A). We designed a primer pair (KEAP1-mut-fwd and KEAP1-mut-rvs) that specifically amplifies mutant *KEAP1* and 2 primer pairs that amplify 5' or 3' portion flanking the mutation of *KEAP1* (KEAP1-N-fwd and KEAP-N-rvs, or KEAP1-C-fwd and KEAP1-C-rvs, respectively). It was found that expression of the c.879_880delinsA mutant was almost nil in the thyroid tissues of the patient as well as in the normal thyroid (Fig 4B, upper panel). On the other hand, expression of the 5' and 3' portion of *KEAP1* were observed in the thyroid of patient; however, the expression level was lower in the tissues of patient than normal thyroid (Fig. 4B, second and third panels). These results suggested that truncated KEAP1 protein is not generated probably due to non-sense mediated mRNA decay, and that the expression of wild type KEAP1 protein might be decreased due to low transcription product, at least in the thyroid tissues of the patient.

**Figure 5:**
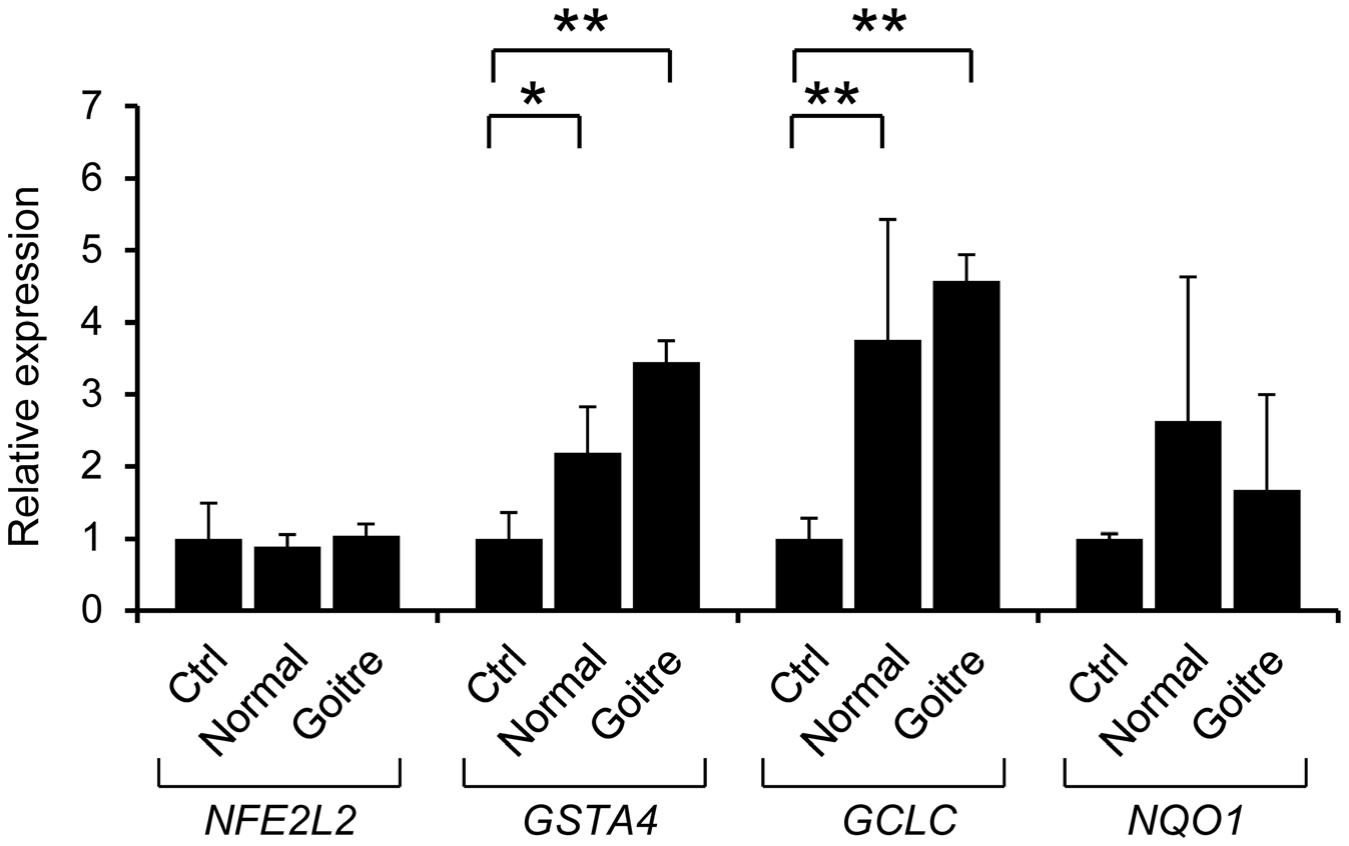
Up-regulation of NFE2L2 target genes in the thyroid tissues of the patient. Results of the quantitative PCR of *NFE2L2* and its target genes *GSTA4*, *GCLC* and *NQO1* in the normal and goitre region of thyroid tissues obtained from the proband. The expression levels relative to control RNA from an adult thyroid are shown. The significance with the t-test is indicated with asterisks (*p<0.05 and **p<0.01).

To investigate the effect of decreased expression of *KEAP1*, we performed qPCR analysis for the NFE2L2 target genes, *GCLC*, *GSTA4* and *NQO1*. As shown in Figure 5, the expression of *GCLC* and *GSTA4* were significantly elevated in both the normal and goitre portion of the thyroid tissue obtained from the proband compared with control thyroid RNA. Regarding *NQO1*, an increasing trend was detected, but this increase was not statistically significant. The expression of *NFE2L2* was not changed, consistent with the theory that KEAP1 affects NFE2L2 localisation but not expression. The results suggested that the decreased expression of *KEAP1* might result in activation of NFE2L2 target genes, at least in the thyroid of the patients.

## Discussion

The thyroid gland maintains its homeostasis in the face of an increased burden of free radicals and reactive oxygen species produced during iodine metabolism for thyroid hormone synthesis. When the gland is exposed to additional oxidative stress caused by smoking and iodine deficiency, it is assumed that the excessive damage of lipids, proteins and DNA leads to thyroid dysfunction, including goitre formation. Indeed, these environmental factors are well known risk factors for MNG. Therefore, the genes involved in the signal cascades protecting cellular homeostasis against oxidative stress would be candidates for the genetic aetiology of MNG.

The KEAP1-NFE2L2 regulatory pathway is a major biological defence system against oxidative damage [32], [33]. In response to oxidative and electrophilic stress, key cysteine residues of KEAP1 are oxidised to allow NFE2L2 to dissociate from the ubiquitination activity of the complex. NFE2L2 subsequently translocates into the nucleus and transactivates a series of cytoprotective genes, including *NQO1*, *GCLC* and *GST*. Notably, somatic mutations that stabilise NFE2L2, thus conferring protection against oxidative stress, have been identified in *KEAP1* in human cancers [32]. To our knowledge, however, germline mutation in *KEAP1* has not been described in familial cancer cases. A recent study suggested that the NFE2L2-mediated adaptation to oxidative stress is crucial for the early stages of tumour development [34]. In this study, we observed that the expression of NFE2L2 target genes were increased accompanied by decreased expression of wild type *KEAP1* in the thyroid tissues obtained from the proband. This suggests that the KEAP1-NFE2L2 pathway might be impaired in the tissues of the patient with the heterozygous c.879_880delinsA mutation, supporting that this MNG family showed dominant mode of inheritance.

The mechanisms through which the patients in the present study were affected with only MNG are unclear. One possibility is that the thyroid gland is most susceptible to additional oxidative stress, in other words, dysregulated NFE2L2 activation, because this gland is physiologically and continuously exposed to stress during hormone synthesis. It is also possible that tumours developed in multiple organs but were not clinically detected due to insufficient examination. An intensive follow-up of the course of the patients would be required for the early diagnosis of tumours in different organs.

Several linkage loci in addition to chromosome 19 have been detected for familial MNG; therefore, it cannot be concluded that the *KEAP1* is the major causative gene of familial cases. Indeed, this is the first report of *KEAP1* mutation in thyroid diseases. However, the association of common variants of *KEAP1* and *NFE2L2* with sporadic cases of MNG should be considered.

In summary, we identified a new MNG locus on chromosome 19p13.2-q12 through linkage analysis using high-density SNP markers. Subsequent sequence analysis focused on the linkage region revealed a heterozygous mutation of c.879_880delinsA, p.Asp294Thr, fs*23 in *KEAP1*, which plays a central role in cell protection against oxidative stress. In agreement with the molecular function of KEAP1, the NFE2L2 target genes *GSTA4* and *GCLC* were up-regulated in the thyroid tissues of the patient, which may be caused by low expression of wilt type *KEAP1*. The present study implies that a functional defect of genes involved in maintaining the homeostasis against the oxidative stress physiologically generated during thyroid hormone synthesis could cause tumorigenesis in this organ.

## Supporting Information

Figure S1 **Genotype quality check of SNPs for the genome-wide linkage analysis.**
(TIFF)

Figure S2 **Summary of sequencing and read mapping.**
(TIFF)

Figure S3 **Results of the estimated proportions of IBD.** The degree of relatedness for each pair is described to the left of the diagonal (UR: unrelated), and the pairwise proportion of IBD calculated by PLINK is shown to the right of the diagonal. The red, pink, blue and white cells in the proportions of IBD indicate 1st-, 2nd- and 3rd-degree relatives and unrelated pairs, respectively. The results indicated that the proportions of IBD are consistent with the clinically obtained pedigree chart.
(TIFF)

Figure S4 **Co-segregation of the KEAP1 mutation in a multinodular goitre family.** WT/WT: homozygous for wild-type KEAP1, WT/mut: heterozygous for the KEAP1 c.879_880delinsA mutation.
(TIFF)

Table S1 **Clinical features of the patients.**
(DOCX)

Table S2 **Call rate of each sample in the genotyping using Illumina Human CNV370K-Quad Array.**
(DOCX)

Table S3 **Number of SNPs on each chromosome analyzed in the multipoint linkage analysis using Genehunter v2.1r5 program.**
(DOCX)
