# Supplementary figures and images for: Identification of a KEAP1 Germline Mutation in a Family with Multinodular Goitre
Source: PLoS One. 2013 May 28;8(5):e65141. doi: 10.1371/journal.pone.0065141 (PMC3665763; doi:10.1371/journal.pone.0065141)

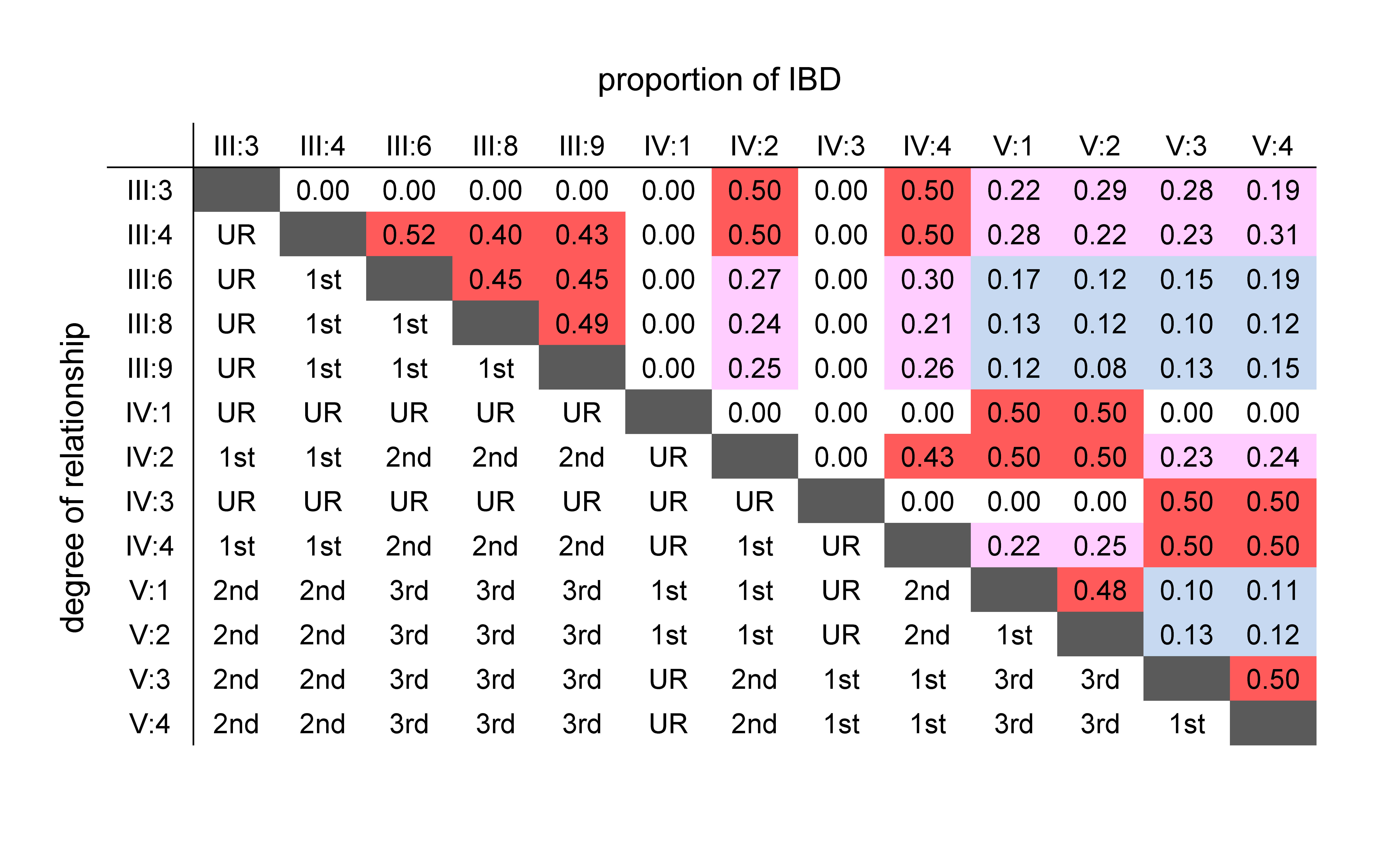

Supplement: Figure S3 — Results of the estimated proportions of IBD. The degree of relatedness for each pair is described to the left of the diagonal (UR: unrelated), and the pairwise proportion of IBD calculated by PLINK is shown to the right of the diagonal. The red, pink, blue and white cells in the proportions of IBD indicate 1st-, 2nd- and 3rd-degree relatives and unrelated pairs, respectively. The results indicated that the proportions of IBD are consistent with the clinically obtained pedigree chart. (TIFF) [file pone.0065141.s003.tiff]
