# Supplementary material for: Identification of a KEAP1 Germline Mutation in a Family with Multinodular Goitre
Source: PLoS One. 2013 May 28;8(5):e65141. doi: 10.1371/journal.pone.0065141 (PMC3665763; doi:10.1371/journal.pone.0065141)
Supplement: Table S1 — Clinical features of the patients. (DOCX) [file pone.0065141.s005.docx]

| Patient | Sex | Onset age | Treatment | Pathological Diagnosis |
| --- | --- | --- | --- | --- |
| III: 4 | F | 50 | Total thyroidectomy | NA |
| III: 6 | F | NA | NA | NA |
| III: 8 | F | 43 | Total thyroidectomy | NA |
| IV: 2 | F | 14 | 14yr: Lt. lobectomy | NA |
|  |  |  | 23yr: Rt. Tumorectomy |  |
|  |  |  | 30yr: Rt.partial lobectomy |  |
| IV: 4 | F | 26 | Rt. lobectomy | NA |
| V: 1 | F | 8 | Lt. lobectomy | Adenomatous goiter |
| V: 2 | F | 15 | Lobectomy | Adenomatous goiter |
| V: 4 | M | 15 | Lobectomy | Adenomatous goiter |

**Supplementary Table 1** Clinical features of the patients
