# Supplementary material for: Identification of a KEAP1 Germline Mutation in a Family with Multinodular Goitre
Source: PLoS One. 2013 May 28;8(5):e65141. doi: 10.1371/journal.pone.0065141 (PMC3665763; doi:10.1371/journal.pone.0065141)
Supplement: Table S2 — Call rate of each sample in the genotyping using Illumina Human CNV370K-Quad Array. (DOCX) [file pone.0065141.s006.docx]

**Supplementary Table 2**

Call rate of each sample in the genotyping using Illumina Human CNV370K-Quad Array

| sample | call rate |
| --- | --- |
| III:3 | 0.9985 |
| III:4 | 0.9979 |
| III:6 | 0.9981 |
| III:8 | 0.9981 |
| III:9 | 0.9980 |
| IV:1 | 0.9983 |
| IV:2 | 0.9978 |
| IV:3 | 0.9977 |
| IV:4 | 0.9976 |
| V:1 | 0.9980 |
| V:2 | 0.9976 |
| V:3 | 0.9976 |
| V:4 | 0.9979 |
| mean | 0.9979 |
