# Supplementary material for: Identification of a KEAP1 Germline Mutation in a Family with Multinodular Goitre
Source: PLoS One. 2013 May 28;8(5):e65141. doi: 10.1371/journal.pone.0065141 (PMC3665763; doi:10.1371/journal.pone.0065141)
Supplement: Table S3 — Number of SNPs on each chromosome analyzed in the multipoint linkage analysis using Genehunter v2.1r5 program. (DOCX) [file pone.0065141.s007.docx]

**Supplementary Table 3**

Number of SNPs on each chromosome analyzed in the multipoint linkage analysis

using Genehunter v2.1r5 program.

| Chromosome |  | Number of SNPs | |  | Chromosome |  | Number of SNPs | |
| --- | --- | --- | --- | --- | --- | --- | --- | --- |
|  |  | 1.0 cM | 0.2 cM |  |  |  | 1.0 cM | 0.2 cM |
| 1 |  | 280 | n.t. |  | 13 |  | 109 | n.t. |
| 2 |  | 270 | n.t. |  | 14 |  | 130 | n.t. |
| 3 |  | 221 | n.t. |  | 15 |  | 115 | n.t. |
| 4 |  | 212 | n.t. |  | 16 |  | 126 | n.t. |
| 5 |  | 196 | n.t. |  | 17 |  | 145 | n.t. |
| 6 |  | 189 | n.t. |  | 18 |  | 121 | n.t. |
| 7 |  | 177 | n.t. |  | 19 |  | 102 | 454 |
| 8 |  | 162 | n.t. |  | 20 |  | 105 | n.t. |
| 9 |  | 163 | n.t. |  | 21 |  | 55 | n.t. |
| 10 |  | 172 | n.t. |  | 22 |  | 62 | n.t. |
| 11 |  | 145 | n.t. |  | X |  | 90 | n.t. |
| 12 |  | 168 | n.t. |  |  |  |  |  |
